# Knowledge and extractivism of *Stryphnodendron rotundifolium* Mart. in a local community of the Brazilian Savanna, Northeastern Brazil

**DOI:** 10.1186/1746-4269-10-64

**Published:** 2014-09-09

**Authors:** Ivanilda Soares Feitosa, Ulysses Paulino Albuquerque, Júlio Marcelino Monteiro

**Affiliations:** Laboratório de Etnobiologia Aplicada e Teórica, Departamento de Biologia, Universidade Federal Rural de Pernambuco, Recife, Brazil; Departamento de Biologia, Universidade Federal do Piauí, Campus Amílcar F, Sobral, Floriano Brazil

**Keywords:** Traditional botanical knowledge, Ethnobotany, Medicinal plants, Plant conservation

## Abstract

**Background:**

This study aims to understand how the stem bark of *Stryphnodendron rotundifolium* Mart. is used by a rural community in the savanna of Northeastern Brazil, associated with a preliminary assessment involving plant population structure and extractivism in the main sites of collection.

**Methods:**

A population structure study and analysis of bark extractivism was conducted in two sites: one within the forest and another at its edge. We had the intention of testing whether there are differences between these sites; since the local extractive practice is prohibited, expecting more intense extraction in the forest interior than its edge by the local fiscalization. We interviewed 120 informants who reported knowing and using the species, and also the places of extractivism. We also calculated quantitative measures of local knowledge, and the influence of gender and age on the knowledge about this species.

**Results:**

Knowledge of the uses was evenly distributed between men and women. A total of 28 specimens were recorded at Site 1, whereas 23 were identified at Site 2, with the specimens at both sites distributed in 4-diameter classes with 4-cm intervals. Nine of the specimens found in Site 1 (32.14%) showed some sign of extraction. No specimen from Site 2 showed signs of extraction. In Site 1, the total area of stem bark removed was 43,468 cm^2^, and the total area of stem bark available was 33,200 cm^2^. In Site 2, only the available stem-bark area of 44,666 cm^2^ was identified because no specimens were harvested. There is no difference in knowledge of this species regarding the gender and age.

**Conclusions:**

*Stryphnodendron rotundifolium* is a key resource for the studied community. A large proportion of bark collected from the first diameter size class may affect the growth of these individuals and may influence the recruitment process. Perhaps, this effect may explain the absence of individuals in some size classes.

## Background

The Cerrado (Brazilian savanna) occupies an area of approximately 2 million km^2^ in the Brazilian territory and is recognized for its high biodiversity [1–5], which has been lost over the years [4]. The increase in the extraction of non-timber forest products stands out among the numerous factors responsible for that loss [6]. Plants used in traditionals communities for several types of applications are found among the plethora of plant resources available in the Cerrado, particularly those used for medicinal purposes [7].

Some of the many medicinal species of the Cerrado are important due to their economic value. However, not all species receive the same attention. In general, people recognize different properties and qualities of particular plant species because different species satisfy different needs [8]. In this sense, the genus *Stryphnodendron* Mart.(“barbatimão”) is known for its numerous uses: the extracts from the stem bark are rich in tannins [9] and are used for curing various diseases, including leukorrhea, diarrhea, inflammatory processes, hemorrhages, hemorrhoids, conjunctivitis [10], malaria, fever, liver disorders, gonorrhea and urethritis [11], and wound healing [12]. The medicinal uses recognized for this genus are largely derived from biological activity studies, and not through ethnobotanical studies.

However, there are few published studies describing the knowledge about the use of plants of extractive importance, such as *Stryphnodendron rotundifolium,* in local communities within the Cerrado despite their relevance as a medicinal resource [6]. In addition, few studies have evaluated the extraction of stem bark from the species of this genus even though it is the main resource collected by the local communities. Borges Filho and Felfili [13] studied only the extraction of bark of populations of *S. adstringens* (Mart.) Coville in four fully protected conservation units, all located in the Paranoá basin, Federal District. According to the authors, all the specimens over 23 cm in diameter showed signs of extraction, and 25-58% of the extracted specimens were used for trade.

Studying about community uses is important because the way in which humans interact with their natural resources is dependent on their subsistence and cultural needs [8]. Ferreira Junior et al. [14] found that certain plant species may have larger areas of bark collected than others depending on community preferences. Plants that are considered to be more versatile, which have a greater diversity of uses, are harvested more often. Such techniques or tools that quantify versatility and diversity of uses associated these variables with the extraction of useful species by local communities. Thus, generalizations or interpretations of effective use of resources should be taken with caution. The use of techniques that quantify knowledge, such as diversity indices, evenness of the informant [15, 16], and the use value [17–19], are selected for their practicality, speed, and ease of use, and fit in the concept of techniques called “consensus of informants” [20, 21]. However, they are unable to estimate the effective use of plant species [21–23]. Gaugris and Van Rooyen [22] have ratified the above information in a study on the use of wood in home construction in Maputaland, South Africa. They found significant distinctions when comparing the results of interviews, where only knowledge is captured, with field inventories, from where wood used in homes was sampled and properly identified, characterizing their practical use.

This paper, taking as model a medicinal species of great local importance, aimed at understanding how people know the resource and at the same time signaling, even approximately, how plant populations are being exploited locally. Thus, this study aimed at analyzing the knowledge about the uses of “barbatimão” in a local community in northeastern Brazil, as well as evaluating the local collection of stem barks. The study also assumes, as a premise, that the collection and use of this resource is a common practice in the region studied. Lozano et al. [24] in a study conducted at the same site as this research have documented that the “barbatimão” is an important plant in the local market. The authors found that 90.7% of the respondents indicated the region as a collection site for the species.

For this, we analyzed the cultural importance of *Stryphnodendron rotundifolium* Mart. to men and women based on their medicinal uses, plant part usage, and diversity of uses as reported locally. By the fact that interaction with plant resources can be established in different ways between men and women, we study the influence of gender on the local knowledge of this species. According to some authors, the knowledge of medicinal plants is greater among women than among men [25, 26]. This trend was observed when the number of known medicinal plants among different genders and ages was evaluated. Regarding the age factor, Silva et al. [25] claim that older people have more knowledge, because they have more experience and contact with these plant resources. However, it is noted that the species that are known and preferred for use, either by men or women of different ages, tends to suffer greater-use pressure [14].

## Materials and methods

### Study site

The study was conducted in the municipality of Jardim, which has approximately 25,853 inhabitants, 7,910 living in the urban area and 17,943 in the rural area; the latter segment is composed of 12,727 men and 13,094 women [27]. The municipality is located 435 km from the state capital of Ceará, Fortaleza, in NE Brazil. The climate is subhumid hot tropical and mild hot semi-arid tropical, with average temperatures ranging from 22°C to 24°C and with 790.4 mm annual rainfall; the rainy season is from January to May [28].

The selected rural community is known as Horizonte and is located 14 km from the urban center of the municipality of Jardim and approximately 8 km from Araripe National Forest (Floresta Nacional do Araripe, FLONA). This community was selected because it is responsible for most of the extraction resources of the Araripe National Forest when compared with other communities. According to data from the local health center, the community has 1,120 inhabitants, among whom 463 are of age 18 years or older. The community is structured in a main street, where houses are immediately adjacent to each other, and properties that are further away have a great distance between them.

**Figure 1:**
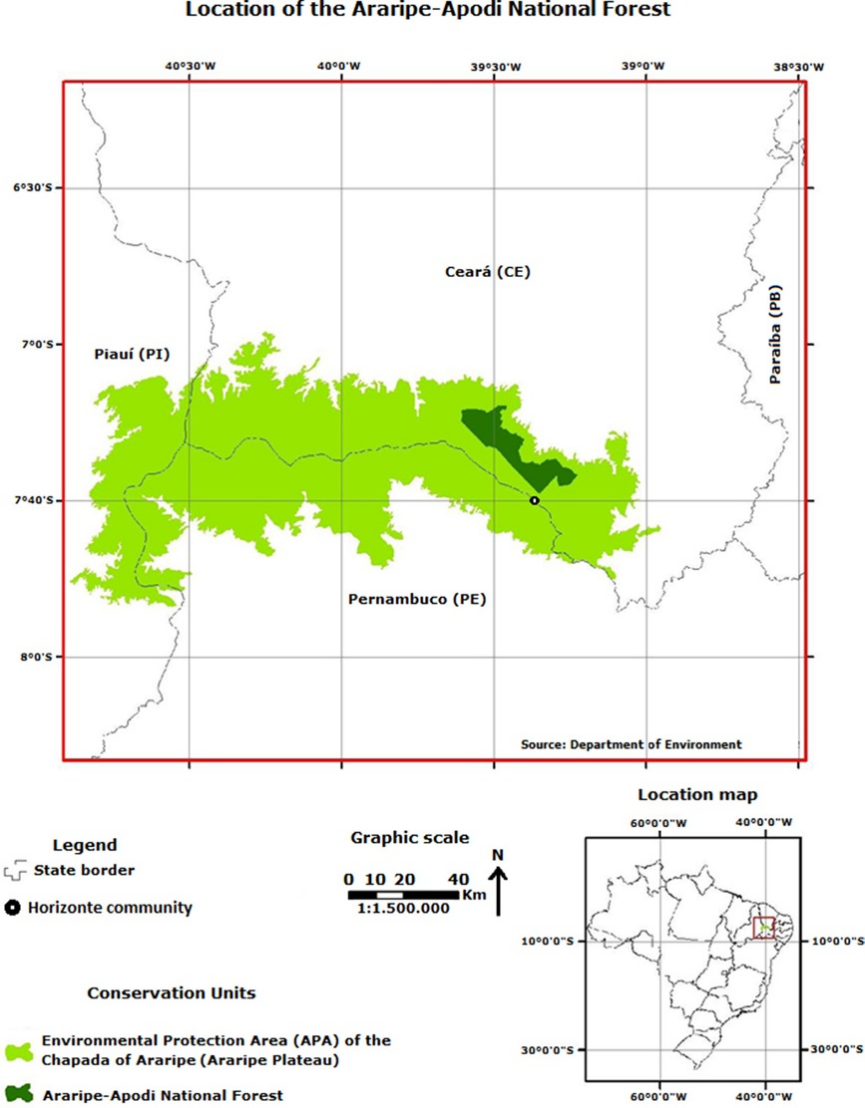
**Location of the Araripe National Forest (Floresta Nacional do Araripe, FLONA), Ceará (NE Brazil).** Source: Department of Environment (Ministério do Meio Ambiente) [4].

The extractivism of *Stryphnodendron rotundifolium* stem bark conducted by the community was evaluated in the Cerrado (like savanna vegetation), which encompasses an area of 16,327 ha within FLONA [29], representing 42.67% of the total area (38,262 ha) (Figure 1), in the state of Ceará. This species was selected because, within the group of medicinal plants extracted by the community, it is considered as one of the most important species.

### Ethnobotanical research

This study was submitted to the ethics committee (CEP) and approved under number CAAE-0270.0.172.000-11 by the CEP of the Center for Health Sciences of the Federal University of Pernambuco. To carry out this research in the Araripe National Forest, the study was also approved (N. 26900–1) by the SISBIO, which is the System for Authorization and Information on Biodiversity of the Chico Mendes Institute for Biodiversity Conservation (ICMBio), the agency in charge of managing FLONA-Araripe.

The researchers sought to gradually establish a friendly and trustworthy relationship with the informants, which is the usual procedure in ethnobotanical studies. The need for that relationship is reinforced given the reports of resource extraction in FLONA, which is a region protected by the federal government where harvesting is not permitted, except for pequí (*Caryocar coriaceum* Wittm.), which is restricted to a given season, and the collection of dry wood, which is restricted to once a week.

The study was conducted between November 2010 and May 2011. Among the total adult population, 150 people were randomly selected for the study. Semi-structured interviews were conducted [30] to identify the knowledge of use of “barbatimão” among informants.

Issues related to the uses, plant part used, forms of use, site, preferential harvesting season, and possible substitutes for “barbatimão” were addressed. Each interview was held with a single person, and the interview length varied according to each informant’s experience. The data gathered from each informant through the interviews were entered into spreadsheets and split into categories for subsequent analysis. This age cutoff was assigned based on what has been used in previous studies [30–33].

### Population structure and stem-bark extraction from *Stryphnodendron rotundifolium*Mart

The structure of *S. rotundifolium* was evaluated in two plots defined at two different sites. The areas used for population structure and extraction studies were identified by the extractors and located near the community.

Both plots were split into 100 continuous divided subplots of 10 × 10 m, totaling an area of 2 ha. All the *S. rotundifolium* specimens with the diameter at ground level equal to or greater than 3 cm were measured in terms of the circumference at ground level and circumference at breast height. Only live specimens were analyzed for this study, sampling a total of 28 specimens in Site 1 and 23 specimens in Site 2.

**Figure 2:**
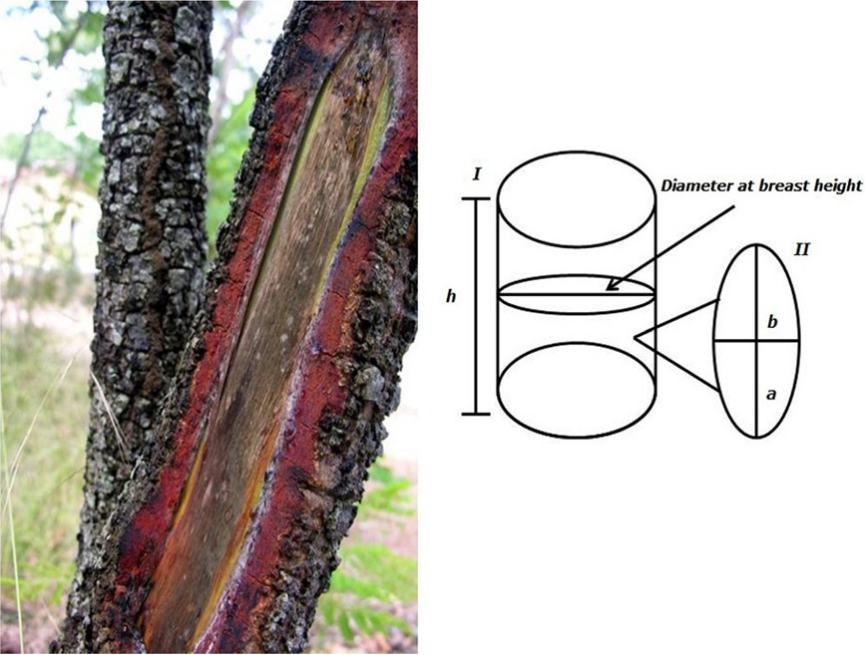
**Measurements of the area of available and harvested stem bark.** I: the cylinder represents the plant stem, and the height (h) and diameter at breast height (DBH) were measured. II: the ellipse represents a section of harvested stem bark, and the length **(a)** and width **(b)** were measured. Adapted from [14].

The areas of stem-bark extraction from the specimens were assessed by adapting the method of Ando et al. [34], wherein the scars’ length (a) and height (b) were measured (Figure 2) by calculating the area of an ellipse (3.14 × a × b) [14].

The visual classification system described by [35] was used to analyze the levels of damage per specimen based on the percentage of stem-bark extraction in a 2-m range from ground level, and the levels of use were classified into seven categories: 0, without damage; 1, less than 10% stem-bark extraction; 2, 11-25% stem-bark extraction; 3, 26-50% stem-bark extraction; 4, 51-75% stem-bark extraction; 5, 76-100% stem-bark extraction; 6, 100% stem-bark extraction (causing the death of the specimen) (adapted from Borges and Felfili [13]).

The available stem-bark area was calculated to determine how much stem bark the *S. rotundifolium* specimens are able to produce. That measurement enabled the inference of the amount of stem bark available to harvesters. That calculation measures the area of stem bark that may be generated in a 2-m height (*h*) range, rather than the total amount of stem bark available for extraction at the time.

For this purpose, the diameters at breast height (DBH) were measured at 1.30 m, assessing the area by calculating (3.14 × DBH × *h*) (Figure 2).

Samples from different *S. rotundifolium* specimens were collected, herborized according to Mori [36], and incorporated into the Dárdano de Andrade Lima Herbarium of Agronomic Institute of Pernambuco (Instituto Agronômico de Pernambuco, IPA).

### Data analysis

Resource availability was calculated based on the population structure of the species. We also evaluated the extraction of bark based on the percentage of bark extracted, the area of removed bark, and the availability of individuals of *Stryphnodendron rotundifolium* Mart. The cultural importance of this species was used to infer how knowledge about the uses of *S. rotundifolium* Mart. was connected to ecological data. The gender analysis was based only on interviewee knowledge about plant usage to identify which sex had more plant usage knowledge because it is known that men and women play different roles in the community.

Local harvester knowledge about “barbatimão” was analyzed using quantitative measurements [31]. The knowledge regarding the use of “barbatimão” among men and women from different ages was analyzed to assess whether those factors affected the knowledge about the species. A comparative analysis of the informants’ knowledge based on their age and gender was performed based on two quantitative measures: the informant diversity value (IDV) and informant equitability value (IEV) [15]. The IDV index represents the number of use-citations given by an interviewee (Ux) divided by the total number of uses. This metric measures how many interviewees used a given species and how the knowledge of the plant was distributed among the interviewees. The IEV was defined as each informant’s diversity value (IDV) divided by the highest informant diversity index found (IDVmax). This variable measured the degree of homogeneity among interviewee knowledge. Significant differences in terms of gender and age were tested using a Kruskal-Wallis test at 5% probability. All the analyses were processed using the BioEstat 5.0 software [37]. All the informants were grouped according to age and gender classes into adults ≥ 40 years of age and < 40 years of age [30]; women 40 years or older (n = 44); women younger than 40 years (n = 21); men 40 years or older (n = 32); and men younger than 40 years (n = 23).

Other analyses were used to assess the knowledge and use of each species, but these were not analyzed based on gender. The index included see [15, 31] the PC index (purpose consensus value). The PC index is the number of registered reports of each use divided by the total number of reports for all uses. This index measures the importance of each use and how each of them has contributed to the local use.

The data were analyzed to assess the stem-bark extraction from specimens of *S. rotundifolium* in the vegetation as follows: the species’ population structure was distributed into diameter classes, which were established at 4-cm intervals. Accordingly, the specimens were distributed in the following classes: 1 (0–4 cm); 2 (4.1-8 cm); 3 (8.1-12 cm); and 4 (12.1-16 cm).

The levels of extraction damages were analyzed based on the distribution of the percentages of stem-bark extraction from each of the specimens per diameter class, according to the following classes: 1 (0–4 cm); 2 (4.1-8 cm); 3 (8.1-12 cm); 4 (12.1-16 cm); 5 (16.1-20 cm); 6 (20.1-24 cm). A Spearman’s correlation test was performed to assess the harvesters’ criterion of specimen selection for stem-bark extraction, correlating the level of extraction with the diameter of the harvested specimens. To analyze the area of stem bark extracted from the specimens by the harvesters, the means of stem-bark extraction from the specimens according to the diameter classes were calculated. To analyze the area of available stem bark, which is represented by the amount of stem bark that the specimen is able to generate in a 2-m height range, the means of the available stem-bark area of the specimens per diameter class were calculated, similar to the calculation of the area of stem-bark extraction [34].

## Results

### Knowledge of *Stryphnodendron rotundifolium*Mart

*S. rotundifolium* is a widely known species in the Horizonte community. All of the interviewees (55 men and 65 women) reported that they knew about the plant and had already used the plant at least once for treating a disease. Only 3 informants reported ignorance of the medicinal use of “barbatimão,” although these respondents were aware that the plant was commonly used in the community.

According to IDV and IEV (Table 1), the knowledge of uses of *Stryphnodendron rotundifolium* was evenly distributed between men and women of different age groups. No significant differences were found between the values of the gender or the age groups when assessing whether knowledge was homogeneous between men and women; however, some uses were reported by a reduced number of interviewees, indicating that a number of interviewees were more knowledgeable than others.

**Table 1:** Mea**surements of knowledge regarding the species** ***Stryphnodendron rotundifolium*** **Mart.**

Variables	Values
Total informants	120
Number of reported uses	206
Use purposes	40
**Measures**	**Average and standard deviation (X ± SD)**
Total IDV	0.043 ± 0.021
Total IDV women	0.044 ± 0.021 a
IDV women < 40 years of age	0.042 ± 0.023 a
IDV women ≥ 40 years of age	0.044 ± 0.021 a
Total IDV men	0.042 ± 0.021 a
IDV men < 40 years of age	0.040 ± 0.020 a
IDV men ≥ 40 years of age	0.045 ± 0.022 a
Total IEV	0.436 ± 0.217
Total IEV women	0.442 ± 0.219 a
IEV women < 40 years of age	0.428 ± 0.239 a
IEV women ≥ 40 years of age	0.448 ± 0.212 a
Total IEV men	0.429 ± 0.215 a
IEV men < 40 years of age	0.402 ± 0.209 a
IEV men ≥ 40 years of age	0.450 ± 0.221 a

X = mean; SD = standard deviation.

The letter “a” in the column indicates nonsignificant values at 5%. IDV = informant diversity value; IEV = informant equitability value.

No significant differences were found between age categories. The data showed that older men were not more knowledgeable than younger men (H = 0.50; p = 0.47) and that older women were not more knowledgeable than younger women (H = 0.15; p = 0.69).

In the Horizonte community, there is a division of labor between men and women. Men are responsible for collecting forest products, and women are responsible for helping with agriculture. Despite the differentiation in daily tasks between genders and the possible loss of interaction between the younger and older community members, knowledge of “barbatimão” was similar across all the analyzed groups.

All (100%) the informants reported that the only utility of “barbatimão” was medicinal, attributing 39 therapeutic indications to the species (Table 2) when questioned about how many different uses “barbatimão” had. The most commonly reported medicinal use of “barbatimão” in the Horizonte community was for “wounds,” receiving a consensus value of 0.16, followed by “ulcer” with 0.15, and “general inflammation” with 0.12.

**Table 2:** **Purpose consensus value of the informants regarding** ***Stryphnodendron rotundifolium*** **Mart.**

Therapeutic indications	Local indications purpose consensus value (PC)
Wound	0.160
Ulcer	0.150
General inflammation	0.120
Headache	0.075
General wound healing	0.054
General internal inflammation	0.050
Gastritis	0.030
Cancer	0.025
Fever	0.025
Leg pain	0.025
Cough	0.025
Cuts	0.025
Body pain	0.010
Inflammation in women	0.010
Skin inflammation	0.010
Scabs	0.010
Flu	0.010
Sore throat	0.010
Heart	0.008
Childbirth inflammation	0.008
Blood pressure	0.008
Blood disorder	0.008
Kidneys	0.008
Lung inflammation	0.005
Sinus infection	0.005
Belly pain	0.004
Urinary infection	0.004
Excessive menstruation	0.004
Inflammation in cuts	0.004
Stomach pain	0.004
Prostate	0.004
Heartburn	0.004
Itch	0.004
Vaginal discharge	0.004
Intestinal inflammation	0.004
Stanch blood from cuts	0.002
Skin allergy	0.002
Swelling	0.002
Tightening the vagina for sexual intercourse	0.002

The analysis was based on the list of disease cited. Horizonte community, NE, Brazil.

All the informants indicated the stem bark as the only structure extracted from the plant, for all kinds of uses.

Regarding the harvesting season, most of the informants (85%) reported the absence of a preferential season for harvesting “barbatimão” stem bark, in contrast to 9% of the informants who reported rainy season as optimal for harvesting stem bark and 3% of the informants who reported ignoring the existence of a harvesting season.

The interviewees indicated no preferred time for bark collection and stated that the extraction of bark only occurred when there was a health problem. The collection of this resource in the Horizonte community is almost exclusively on a domestic scale. Based on this demand, the collection frequency is low; however, informers reported collecting large amounts of bark per event for stocking purposes.

### Population structure and stem-bark extraction of *Stryphnodendron rotundifolium*Mart

**Figure 3:**
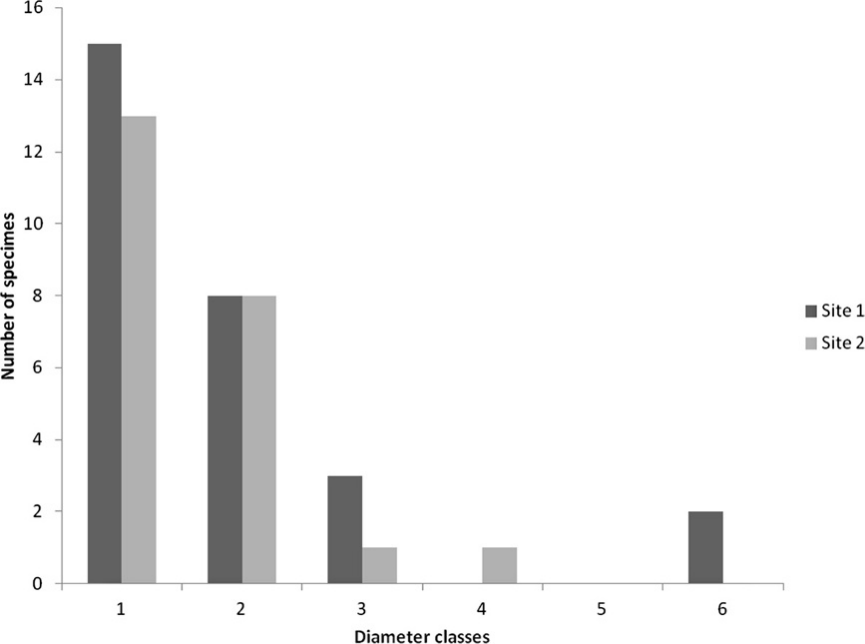
**Distribution of** ***Stryphnodendron rotundifolium*** **Mart. specimens in two Cerrado (Brazilian Savanna) regions in the National Forest of Araripe (Floresta Nacional do Araripe, Ceará, Northeastern Brazil) in diameter classes of 4-cm intervals: 1 (0–4 cm); 2 (4.1- 8 cm); 3 (8.1- 12); 4 (12.1 - 16); 5 (16.1-20); 6 (20.1-24).** Site 1 = study site within the forest; Site 2 = study site near the forest edge.

Twenty eight specimens distributed in four diameter classes were observed in area 1, located inside the forest, with a higher concentration in the smaller classes and a drastic decrease with the increase in diameter. There were no specimens in the last diameter class, which most likely corresponded to the harvesters’ preferred class (Figure 3).

A total of 23 live specimens were observed in area 2, located at the forest edge, also concentrated in the smaller diameter classes (Figure 3), albeit without evidence of specimens with signs of extraction. That result may indicate that other factors most likely exist to explain the absence of extraction, despite the greater resource availability in that area.

Nine of the specimens identified showed some sign of extraction, 1 specimen had up to 10% stem-bark extraction, and 8 specimens showed 100% stem-bark extraction. The number of specimens with 100% stem-bark extraction in the population most likely reflect the harvesters’ use, and the decrease of specimens in the last classes most likely occurred because the total extraction of that resource precludes its regeneration. Regarding the stem-bark extraction per diameter class, the first class (0–4 cm) included 1 specimen with 100% stem-bark extraction, and 14 specimens showed no damage (Table 3). Sequentially, in the second class (4.1-8 cm), 4 specimens showed 100% stem-bark extraction, and 5 specimens showed no signs of damage (Table 3). In the third class (8.1-12 cm), 1 specimen had 10% stem-bark extraction, and 2 specimens showed 100% stem-bark extraction (Table 3). No specimens were included in classes 4 (12.1-16 cm) and 5 (16.1-20 cm) (Table 3). The two specimens included in the class ranging from 20.1 to 24 cm showed 100% stem-bark extraction (Table 3).

**Table 3:** **Distribution of the signs of extraction in** ***Stryphnodendron rotundifolium*** **Mart.**

Levels of extraction									
Diameter class (cm)	0	1	2	3	4	5	6	Harvested total	Overall total
	0%	(1-10%)	(11-25%)	(26-50%)	(51-75%)	(76-100%)	100%		
**0-4**	14	0	0	0	0	0	1	**1**	**15**
**4.1-8**	5	0	0	0	0	0	3	**3**	**8**
**8.1-12**	0	1	0	0	0	0	2	**3**	**3**
**12.1-16**	0	0	0	0	0	0	0	**0**	**0**
**16.1-20**	0	0	0	0	0	0	0	**0**	**0**
**20.1-24**	0	0	0	0	0	0	2	**2**	**2**
**Total**	**19**	**1**	**0**	**0**	**0**	**0**	**8**	**9**	**28**

The analysis was based in extraction per diameter class in Cerrado (Brazilian Savanna) region, Araripe National Forest, Northeastern Brazil.

The highest incidence of stem-bark extraction was found in classes 2 (4.1-8 cm) and 3 (8.1-12 cm), which showed that the ratio between harvested specimens and the number of specimens present in the classes increased with the increase in diameter (Table 3), with 100% harvested specimens in the 20.1-24-cm class. The extractors’ preference for harvesting resources from specimens with larger diameters most likely explains the absence of specimens in some of the larger classes.

However, no significant correlation was found among the variables (rs = -0.14, p > 0.05) when the level of damage and diameter of the *S. rotundifolium* specimens were correlated.

**Figure 4:**
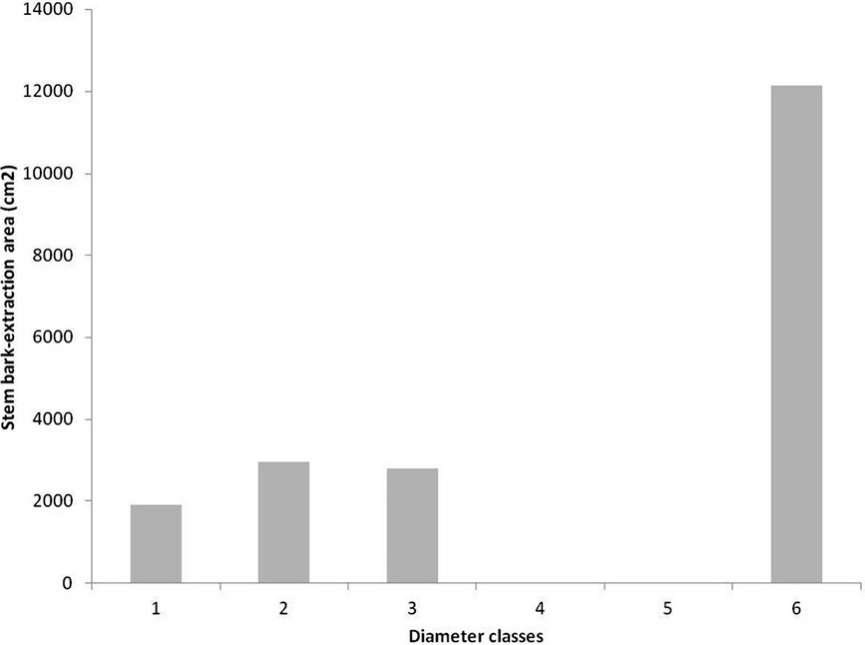
**Area of stem bark extracted from** ***Stryphnodendron rotundifolium*** **Mart. specimens from a region of Cerrado (Brazilian Savanna), Araripe National Forest (Floresta Nacional do Araripe, Ceará, Northeastern Brazil) distributed in diameter classes with 4-cm intervals: 1 (0–4 cm); 2 (4.1- 8 cm); 3 (8.1-12); 4 (12.1-16); 5 (16.1-20); 6 (20.1-24).** Study site within the forest.

The total area of stem-bark extracted from the specimens harvested within the forest was 43,468 cm^2^. The analysis according to the diameter classes shows that the stem–bark extraction area was 1,900 cm^2^ in the first class (0–4 cm), corresponding to a single specimen (Figure 4). The second class (4.1-8 cm), with 3 specimens harvested, showed a mean stem-bark extraction of 2,966 cm^2^, followed by the third class (8.1-12 cm), with 2 harvested specimens, showing a mean of 1,418 cm^2^ (Figure 4). No specimens were included in classes 4 (12.1-16 cm) and 5 (16.1-20 cm) (Figure 4). That absence most likely results from the harvesters’ preference for extracting stem bark from specimens with larger diameters because the amount of stem bark available for harvesting is proportional to the specimen’s size. The highest value was found in the specimens from class 6 (20.1-24 cm), with 12,150 cm^2^; these specimens had all their stem bark extracted (Figure 4). No specimens were harvested at the forest edge.

The total available area of stem bark within the forest was 33,200 cm^2^. Comparing those values with values regarding the stem-bark extraction area (43,468 cm^2^) may suggest that most of the stem bark was extracted from the specimens; however, the value of the available stem-bark area shown herein is underestimated for two reasons. The first reason regards four young specimens without a measurable diameter at breast height at 1.30 m, which is the height required for measuring the available stem-bark area in the methodology used [34], and the second reason regards the number of specimens in the population with 100% stem-bark extraction, wherein the available area of stem bark was also not assessed because the plants no longer had the ability to regenerate the resource for subsequent harvesting.

**Figure 5:**
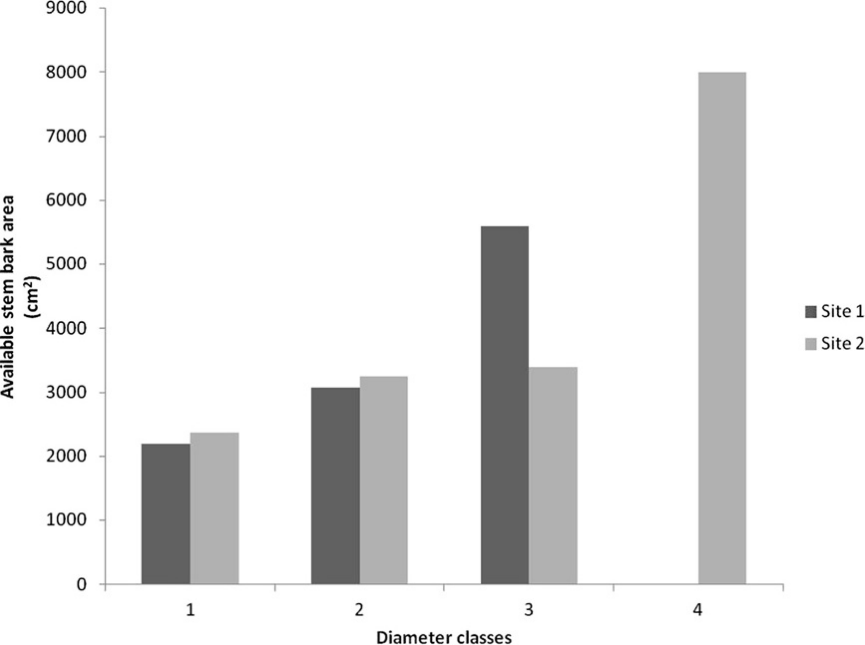
**Available stem-bark area of** ***Stryphnodendron rotundifolium*** **Mart. specimens from two Cerrado (Brazilian Savanna) sites in the Araripe National Forest of (Floresta Nacional do Araripe, Ceará, Northeastern Brazil) in diameter classes with 4-cm intervals: 1 (0–4 cm); 2 (4.1- 8 cm); 3 (8.1-12); 4 (12.1-16).** Site 1 = study site within the forest; Site 2 = study site near the forest edge.

The mean available stem area in the first diameter class (0–4 cm) was 2,200 cm^2^ (Figure 5). The mean in the second class (4.1-8 cm) was 3080 cm^2^ (Figure 5). The highest values were found in the diameter class 3 (8.1-12 cm), with 5600 cm^2^ (Figure 5). No available stem bark was found in class 4 (12.1-16 cm) due to the absence of specimens (Figure 5).

A total available area of stem bark of 44,666 cm^2^ was found in the 23 specimens sampled at the forest edge. No specimens with signs of bark extraction were found in that plot. The 13 specimens found in the first diameter class (0–4 cm) showed a mean of 2,366 cm^2^ (Figure 5), higher than that of the 15 specimens from the same class of Site 1, which is explained by the number of specimens (7) that failed to reach the DBH at 1.30 m in Site 1. The second class (4.1-8 cm), with 8 specimens, showed a mean of 3,255 cm^2^ (Figure 5). Class 3 (8.1-12 cm), with 1 specimen, showed a mean of 3,400 cm^2^ (Figure 5). The same diameter class in Site 1, with 3 specimens, had a mean of 2,789 cm^2^, which included specimens with 100% stem-bark extraction in Site 1. Class 4 (12.1-16 cm), with 1 specimen, showed a stem-bark area of 8,000 cm^2^ (Figure 5), whereas the same class in Site 1 had no specimens.

## Discussion

### Knowledge of *Stryphnodendron rotundifolium*Mart

According to the results shown, knowledge about *Stryphnodendron rotundifolium* Mart. is widely disseminated among the members of the Horizonte community, taking into account both the gender and age factors. Almeida et al. [26], studying medicinal plants in a community located in the Atlantic Forest in northeastern Brazil, found that women had greater knowledge of medicinal plants when compared with men. The authors believe that this fact can be explained by the division of activities performed by the various genders. This finding reinforces the idea that even if there is a separation of the daily tasks between men and women, this fact did not influence the knowledge, demonstrating how this resource is widely known within the community. Therefore, although the Horizonte community provides a setting conducive to this finding, the greater knowledge of medicinal plants by women was not observed. These results were also found by Lins Neto et al. [33] studying *S. tuberosa,* who noted variation in the knowledge of this species between men and women. Despite this species being a food resource but not medicinal as the species cited in this study, what we perceive is the similar knowledge among men and women right across the differentiation of daily tasks. Regarding “barbatimão,” this observation may be explained by the fact that men harvested the plant resource, but it was mostly women who used the plant resource in medicinal preparations. According to Silva et al. [25], women have more knowledge because they are often sought to diagnose and treat diseases; however, in this study, this fact was not observed. Monteiro et al. [16] studying *Myracrodruon urundeuva* (Engl.) Fr. All. found that greater knowledge of medicinal plants was often found among women, both regarding the repertoire of plants known and the diversity of uses attributed to each species. It is observed that the knowledge of the uses of “barbatimão” does not follow the trend found in many other studies [16, 25].

Similar to gender, the age factor did not affect the knowledge of the use of “barbatimão” in the community in question. Most of the men younger than 40 years of age work during the cane-harvesting seasons in Paraná upon reaching adulthood. This distancing from activities performed by the community and the lack of contact with elders would lead to the expectation that younger men have less knowledge than the elders. However, even with this gap in activities of “Horizonte” community, young people remained with a broad knowledge of the uses of “barbatimão,” which may reflect how important this resource is. His pattern was found by Monteiro et al. [16], who noted that the variety of plants known by the youngest study subjects, in the age group 18–28 years, was lowest, which could be explained by the lack of experience and contact with the region’s plant resources. Although the same distancing by young people occurred in the present community, the reports showed that the main survey was indicative of “barbatimão” for wound treatment, and young people’s mothers maintained the habit of treating cuts during their childhood with “barbatimão” stem-bark powder, suggesting that the knowledge had been transferred to individuals well before they reached adulthood.

The number of medicinal uses attributed to “barbatimão” by both gender and age groups and the number of reports of bark use (120) indicate that the community harvests and uses this resource because the species is versatile and can be used to treat a large number of diseases. According to Ferreira Junior et al. [14], species with high versatility are expected to show a higher incidence of harvesting. According to the same author, the use-pressure may also be linked to a given resource. According to the data shown, the stem bark is regarded as the only plant part used for drug preparation in the various use purposes attributed to the plant. Similar situation has been found in some species of the Caatinga [16, 38, 39]. Monteiro et al. [16] noted a consensus between the two communities studied regarding the stem bark as the most commonly used part of *Anadenanthera colubrina* and *Myracrodruon urundeuva* and suggested that this use most likely resulted from the higher levels of polyphenolic extracts contained in that structure. Soldati et al. [39] found a higher number of reports for the use of the stem bark of *A. colubrina* than other structures when analyzing three different sites of resource harvesting, and the harvests differed between the native forest and anthropogenic sites.

The informants indicated “barbatimão” as a preferred plant for the treatment of various diseases. This preference leads in most of those cases to a noticeably higher incidence of harvesting. Ferreira Junior et al. [14] observed from studying the collection of barks in medicinal plants of the Caatinga that individuals with larger areas of barks were collected from plants indicated as preferred. That is, the pressure of use was higher in plants indicated preferred, when compared with the nonpreferred plants [14]. That scenario is supported by the argument that people believe in the efficacy of “barbatimão,” although there are other plants suitable for the same uses; thus, a potential use-pressure may be expected on this species. This pattern was actually observed by Ferreira Junior et al. [14], who noted that plants preferably used for treating inflammation had a larger harvested area.

The absence of a harvesters’ preferred season for harvesting also shows that the extraction of that product may occur throughout the year given the perennial availability of the resource, which could be one of the factors explaining the large proportion of stem bark extracted and the dead specimens in the vegetation, as the constant harvesting precludes stem-bark regeneration.

### Population structure and stem-bark extraction of *Stryphnodendron rotundifolium*Mart

Analysis of the number of specimens per diameter class shows that they are not distributed according to the inverted J model, suggesting that the population is in imbalance. This result contrasts with some other studies performed using the species that report that the specimens are arranged in a stable population capable of self-regenerating [13].

Although there was a low frequency of collection indicated by the interviewees, data on the population structure of the plants showed individual vulnerability. These data showed a diversity of uses attributed to “barbatimão,” demonstrating its versatility. Thus, it can be inferred that the high percentage of bark collected accommodates the demand.

The highest frequency of individuals of stem-bark harvesting was found in the first two diameter classes. Ferreira Junior et al. [14] and Lins Neto et al. [39] similarly found results with specimens of *Anadenathera colubrina* and *Myracrodruon urundeuva*, assessing a higher frequency of specimens harvested in the initial life-cycle classes.

The collection of bark did not follow a pattern because the harvesters tended to extract the resource randomly. However, the extraction of bark from small individuals affects their growth. According to Borges Filho and Felfili [13], bark extraction from small individuals affects their growth because it removes the phloem, thus preventing the sap from flowing. According to Soldati et al. [40], depending on the amount of bark removed and the individual's ability to resist damage, the extraction bark can lead to the death of the plant. Thus, smaller individuals are more likely to not be able to tolerate the impact of collection. A similar situation was found in Lins Neto et al. [33] and Borges Filho and Felfili [13], demonstrating that a lack of criteria for the collection of individuals can lead to extinction if too many small plants are harvested.

Despite the harvest process occurring randomly in the choice of the individual, the data suggest that there was an increased incidence of collection in the last diameter classes, justified by the absence of individuals in some classes, which does not mean that they are more collected in terms of quantity of barks collected. This pattern has been found by other studies on the genus, as reported by Borges Filho and Felfili [13], who noted that 15-cm-diameter classes were preferred for harvesting. The results published showed that the absence of specimens in that population started from that diameter class. As stated previously, the intensity of collection can lead to death of individuals. Additionally, there are ways to extract the resources that severely impair the individual, regardless of the amount of bark collected. As an example, we can mention the girdling of the stem-bark individuals. The absence of specimens in some of the diameter classes found in the populations of *S. rotundifolium* from that study might not have resulted from a species’ biological limitation, according to the comparison of the data found herein and from other studies that identified specimens of *S. adstringens* with up to 31-cm diameter [13]. Furthermore, the presence of specimens with much larger sizes and signs of extraction well above those found in that study was noted in sites identified by the harvesters in the interviews, located in difficult-to-reach regions.

Soldati et al. [38] studied a population of *Anadenathera colubrina* in the Brazilian Northeastern semi-arid region and noted that the greatest number of individual harvestings were of those with the greatest diameters, which could be explained by the harvesters’ belief that stem bark from larger specimens produced stronger remedies, according to the same authors.

The results from this study showed that although the site located within the forest only contained three specimens more than the site located at the forest edge, the presence of signs of extraction was noted in the former site, while no signs of extraction were detected in the latter, thereby indicating that other factors exist that should explain the harvesters’ preference for specimens found in the innermost forest region. Oliveira et al. [41] studied conservation-priority species in two Caatinga regions and unexpectedly found the highest incidence of specimens in the site located far from the community; however, the author argued that the harvesting frequency of a given resource may be closely linked to the resource’s availability, which may explain the results concerning stem-bark harvesting in Site 1 of that study. One of the factors that may explain the harvesting exclusively in Site 1 is the illegality of the stem-bark harvesting practice within FLONA, which often inhibits harvesting in places where surveillance is most effective, redirecting resource extraction to outlying sites. Another factor is the characteristics of Site 2 (forest edge), which may have been a harvesting site in the past, wherein a decrease in resource use was followed by the decline of specimens, leading harvesters to shift the harvesting toward the inner forest. According to Soldati et al. [40], the density of specimens in a habitat most likely affects the number of extraction events performed by harvesters, affecting the harvesters’ behavior when using resources in different habitats. Accordingly, the highest means of stem-bark extraction in Site 1 were found in the classes with the highest number of specimens; thus, the harvesters’ decisions may be based on the greater availability of the resource to be extracted. The strong consensus among the informants, who reported that FLONA was the preferred site for stem-bark harvesting, is another indication that stem-bark harvesting is most likely responsible for the imbalance of those populations.

The resource unavailability of specimens with larger diameters at Site 1 may have intensified the harvesting from small individuals. Similar results were found by Gauoe and Ticktin [42] when studying harvesting patterns of *Khaya senegalensis* stem bark in Benin, Africa, suggesting that the intense pressure placed on large specimens over time led harvesters to extract the resource from specimens with smaller diameters. Similarly, Ferreira Junior et al. [14] studied medicinal species from the Caatinga and found evidence for extraction from *A. colubrina* in the first classes, although with extracted stem-bark values well below those found in this study.

The comparison of the means of the available stem bark between the specimens from both the sites shows that specimens from Site 2 have a stem-bark area similar to that of specimens from Site 1, despite the significantly lower number of specimens. That finding may be explained by the absence of extraction of specimens from Site 2 and the high number of specimens from Site 1 without a DBH at 1.30 m, a measurement required for calculating the available stem bark. That height limitation may have resulted from harvesting, as harvesting may harm plant growth [13].

## Conclusion

We conclude that “barbatimão” (*Stryphnodendron rotundifolium* Mart.) is widely known by the Horizonte community. Because this knowledge is shared by both men and women, independent of the activities that they perform, we conclude that the use of “barbatimão” is widespread, because of the evidence of stem-bark collection.

When collecting bark, harvesters do not discriminate between different diameter classes. A large proportion of bark collected from the first diameter size class may affect the growth of these individuals and may influence the recruitment process. Perhaps, this effect may explain the absence of individuals in some size classes. But additional studies are necessary to address this topic.

A long-term increase in stem-bark extraction may increasingly compromise the species’ population structure, as suggested by the presence of dead specimens and the absence of specimens in some diameter classes, thereby indicating that harvesting practices should follow special guidelines based on the development of conservation strategies and sustainable harvesting.

Barbatimão both as “fava d’anta” (*Dimorphandra gardineriana* Thul.) and “pequi” (*Caryocar coriaceum* Wittm.) are included in a group of commercially important plants in the region, whose extraction provides income to residents of adjacent communities; this extraction is performed in FLONA [24]. Thus, it is justified that strategies for management of the species should not be formulated without community involvement. Policies for sustainable harvesting without including the community are unlikely to succeed; thus, successful strategies require the community members’ awareness of such information and of the entire process to be performed. Therefore, future data on the use and harvesting of stem bark must be combined to facilitate proposals for the conservation of this species.

## References

[1] Felfili JM, Nogueira PE, Silva Júnior MC, Marimon BS, Delitti WBC (2002). Composição Florística e Fitossociológica do Cerrado Sentido Restrito no município de Água Boa-MT. Acta Bot Bras.

[2] Souza CD, Felfili JM (2006). Uso de plantas medicinais na região de Alto Paraíso de Goiás, GO, Brasil. Acta Bot Bras.

[3] Vila Verde GM, Paula JR, Carneiro DM (2003). Levantamento etnobotânico das plantas medicinais do cerrado utilizadas pela população de Mossâmedes (GO). Rev Bras Farmacogn.

[4] Ministério do meio ambiente (2006). Programa Nacional de Conservação e Uso Sustentável do Bioma Cerrado.

[5] Guarim Neto G, Morais RG (2003). Recursos medicinais de espécies do cerrado de Mato Grosso: um estudo bibliográfico. Acta Bot Bras.

[6] Zardo RN (2008). Efeito do impacto da extração do pequi (Caryocar brasiliense) no cerrado do Brasil central.

[7] Macedo FM, Martins GT, Rodrigues CG, Oliveira DA (2007). Triagem fitoquímica do Barbatimão [*Stryphnodendron adstrigens* (Mart) Coville]. Revista Brasileira de Biociências.

[8] Blancas J, Casas A, Salicrup DP, Caballero J, Veja E (2013). Ecological and sócio-cultural factors influencing plant management in Náhuatl communities of the Tehuacán Valley Mexico. J Ethnobiol Ethnomed.

[9] Castro AHF, Paiva R, Alvarenga AA, Vitor SMM (2009). Calogênese e teores de fenóis e taninos totais em barbatimão [*Stryphnodendron adstrigens* (Mart.) Coville]. Ciência e Agrotecnologia.

[10] Souza TM, Severi JA, Silva VYA, Santos E, Pietro RCLR (2007). Bioprospecção de atividade antioxidante e antimicrobiana da casca de *Stryphnodendron adstrigens* (Mart.) Coville (Leguminosae-Mimosoidae). Revista de Ciências Farmacêuticas Básica e Aplicada.

[11] Oliveira ALS, Figueiredo AD (2007). Prospecção Fitoquímica das folhas de *Stryphnodendron adstrigens* (Mart.) Coville (Leguminosae-Mimosoidae). Revista Brasileira de Biociências.

[12] Sanches ACC, Lopes GC, Nakamura CV, Dias Filho BP, Mello JCP (2005). Antioxidant antifungal activies of extracts and condensed tannins from *Stryphnondendron obovatum* Benth. Revista Brasileira de Ciências Farmacêuticas.

[13] Borges Filho HC, Felfili JM (2003). Avaliação dos níveis de extrativismo da casca de barbatimão [*Stryphnodendron adstringens* (Mart.) Coville] no Distrito Federal, Brasil. Revista Árvore.

[14] Ferreira Junior WS, Siqueira CFQ, Albuquerque UP (2011). Plant stem bark extractivism in the Northeast semiarid region of Brasil: A new aport to utilitarian redundancy model. Evidence-Based Complement Altern Med.

[15] Byg A, Balslev H (2001). Diversity and use of palms in Zahamena, eastern Madagascar. Biodivers Conserv.

[16] Monteiro JM, Albuquerque UP, Lins Neto EMF, Araujo EL, Amorim ELC (2006). Use patterns and knowledge of medicinal species among Two rural communities from northeastern Brasil’s semi-arid region. J Ethnopharmacol.

[17] Phillips O, Gentry AH (1993). The useful plants of Tambopata, Peru: I. Statistical hypothesis tests with a new quantitative technique. Econ Bot.

[18] Rossato SC, Leitão Filho H, Begossi A (1999). Ethnobotany of Caiçara of the Atlantic Forest Coast (Brazil). Econ Bot.

[19] Lucena RPF, Araujo EL, Albuquerque UP (2007). Does the local availability of woody caatinga plants (Northeastern Brazil) explain their Use value?. Econ Bot.

[20] Phillips O, Alexiades M (1996). Some Quantitative Methods for Analyzing Ethnobotanical Knowledge. Selected Guidelines for Ethnobotanical Research: A Field Manual.

[21] Silva VA, Nascimento VT, Soldati GT, Medeiros MFT, Albuquerque UP, Silva VA, Nascimento VT, Soldati GT, Medeiros MFT, Albuquerque UP, Albuquerque UP, Cunha LVFC, Lucena RFP, Alves RRN (2014). Techniques for Analysis of Quantitative Ethnobiological Data: Use of Indices. Methods and Techniques in Ethnobiology and Ethnoecology.

[22] Gaugris JY, Van RMW (2006). Questionnaires do not work! A comparison of methods used to evaluate the structure of buildings and wood used in Rural Households, South Africa. Ethnobotany Res Appl.

[23] Hoffman B, Gallaher G (2007). Importance indices in ethnobotany. Ethnobotany Res Appl.

[24] Lozano A, Araujo EL, Medeiros MFT, Albuquerque UP (2014). The apparency hypothesis applied to a local pharmacopoeia in the Brazilian northeast. J Ethnobiol Ethnomed.

[25] Silva FSS, Ramos MA, Hanazaki N, Albuquerque UP (2011). Dynamics of traditional knowledge of medicinal plants in a rural community in the Brazilian semi-arid region. Rev Bras Farmacogn.

[26] Almeida CFCBR, Ramos MA, Silva RRV, Melo JG, Medeiros MFT, Araujo TAS, Almeida ALS, Amorim ELC, Alves RRN, Albuquerque UP (2012). Intracultural Variation in the Knowledge of Medicinal Plants in an Urban–rural Community in the Atlantic Forest from Northeastern Brazil. Evidence-based Complement Altern Med.

[27] **Instituto brasileiro de geografia e estatística (IBGE)** http://www.ibge.gov.br/home/

[28] **Fundação Cearense de Metereologia e Recursos hídricos** http://www.funceme.br/

[29] Lima MF (1983). Mapeamento e Demarcação da Floresta Nacional do Araripe.

[30] Albuquerque UP, Lucena RFP, Lins Neto EMF, Albuquerque UP, Cunha LVFC, Lucena RFP, Alves RRN (2014). Selection of Research Participants. Methods and Techniques in Ethnobiology and Ethnoecology.

[31] Monteiro JM, Lins Neto EMF, Albuquerque UP, Amorim ELC, Araújo EL, Albuquerque UP, Almeida CFCBR, Marins JFA (2005). Medidas Quantitativas Para o Estudo de Conhecimento Local Sobre Plantas Medicinais. Tópicos em Conservação, Etnobotânica e Etnofarmacologia de Plantas Medicinais e Mágicas.

[32] Sousa Junior JR, Albuquerque UP, Peroni N (2013). Traditional Knowledge and Management of *Caryocar coriaceum* Wittm. (Pequi) in the Brazilian Savanna, Northeastern Brazil. Econ Bot.

[33] Lins Neto EMF, Peroni N, Albuquerque UP (2010). Traditional Knowlegde and management of Umbu (*Spondias tuberose*, Anacardiaceae): An endemic species from the semi-arid region of Northeastern Brazil. Econ Bot.

[34] Ando M, Yokota HO, Shibata E (2003). Bark stripping preference of sika deer, *Cervus nippon*, in terms of bark chemical contents. For Ecol Manage.

[35] Cunningham AB (1993). African Medicinal Plants: Setting Priorities at the Interface Between Conservation and Primary Healthcare. People and Plants Working Paper1.

[36] Mori SA, Silva LAM, Lisboa G, Corandin L (1989). Manual de Manejo de Herbário Fanerogâmica.

[37] Ayres M, Ayres Junior M, Ayres DL, Santos AAS (2007). BioEstat 5.0: Aplicações Estatísticas nas Áreas das Ciências Biológicas e Médicas.

[38] Soldati GT, Albuquerque UP (2011). Impact assessment of the harvest of a medicinal plant (*Anadenanthera colubrina* (Vell.) Brenan) by a rural semi-arid community (Pernambuco), northeastern Brazil. J Biodiversity Sci, Ecosystem Serv Manag.

[39] Lins Neto EMF, Ramos MA, Oliveira RLC, Albuquerque UP (2008). The Knowledge and harvesting of *Myracrondruon urundeuva* Allemão by Two Rural Communities in NE Brazil. Funct Ecosystems and Communities.

[40] Soldati GT, Albuquerque UP (2012). A New application for the optimal foraging theory: the extraction of medicinal plants. Evidence-Based Complementary Altern Med.

[41] Oliveira RLC, Lins Neto EMF, Araujo EL, Albuquerque UP (2007). Conservation priorities and populations structure of woody medicinal plants in an area of caatinga vegetation (Pernambuco State, NE Brazil). Environ Monitoring Asses.

[42] Gaoue OG, Ticktin T (2007). Patterns of harvesting foliage and bark from the multipurpose tree *Khaya senegalensis* in Benin: Variation across ecological regions and its impacts on population structure. Biol Conserv.

